# A Case Report and Literature Review on Incidental Morgagni Hernia in Bariatric Patients: To Repair or Not to Repair?

**DOI:** 10.7759/cureus.39950

**Published:** 2023-06-04

**Authors:** Christopher Adereti, Jamal Zahir, Emmanuel Robinson, Jacqueline Pursel, Isam Hamdallah

**Affiliations:** 1 Surgery, Ross University School of Medicine, Miramar, USA; 2 Medicine, Ross University School of Medicine, Miramar, USA; 3 General Surgery, Ascension Saint Agnes, Baltimore, USA; 4 Bariatric Surgery, Ascension Saint Agnes, Baltimore, USA

**Keywords:** obesity, laparoscopy, sleeve gastrectomy, bariatric surgery, morgagni hernia

## Abstract

Morgagni hernia (MH) is a congenital diaphragmatic hernia that is often asymptomatic in adult patients. These defects may be discovered incidentally during the intraoperative period and repaired laparoscopically with tension-free synthetic mesh when surgery is warranted. Presently, there is a dearth of studies addressing incidental MH repair in the setting of concomitant bariatric surgery. As such, there are no clear guidelines as to whether or not asymptomatic hernias found incidentally during bariatric surgery require operative repair. Herein, we present the case of a morbidly obese female patient with an incidental Morgagni defect that was identified during an elective sleeve gastrectomy. We also reviewed the literature to assess the efficacy of concurrent bariatric surgery and hernia repair.

## Introduction

The Morgagni foramina, also known as sternocostal triangles or Larrey’s triangles, are small anterior diaphragmatic defects in which the internal thoracic arteries course to become the superior epigastric arteries, along with associated veins and lymphatics [1]. Protrusion of viscus through these foramina results in Morgagni hernias (MHs). These defects are the rarest manifestation of congenital diaphragmatic hernias (CDHs), comprising 2-3% of cases [2-4]. Adult patients are often asymptomatic, and these defects are often discovered incidentally during diagnostic workup or the intraoperative period [2]. In addition to MHs, hernia defects can also occur within the foramen of Bochdalek, which often occur along the posterolateral wall of the diaphragm [1]. Although more common than Morgagni defects, Bochdalek hernias often present with more severe symptoms such as acute respiratory distress, abdominal pain, and ileus [5]. Nevertheless, multiple studies have recommended surgical repair for both types of defects [2,6-12]. To date, there are a limited number of case reports that describe the surgical management of MHs in obese patients undergoing concomitant bariatric surgery [2,8-12] and fewer studies that evaluate the need for surgical repair of those with incidental MHs [2,9,10]. However, to our knowledge, there are no studies that evaluate the need for surgery in bariatric patients with asymptomatic MHs identified incidentally. In this report, we describe the case of an asymptomatic middle-aged female who was found to have an incidental Morgagni defect while undergoing sleeve gastrectomy.

## Case presentation

A 58-year-old female with a history of morbid obesity, obstructive sleep apnea, dyslipidemia, and hypertension presented to our service at Saint Agnes for a scheduled laparoscopic sleeve gastrectomy with an upper endoscopy. The patient denied having any recent history of surgery, and her review of systems was negative for chest pain, palpitations, cough, wheezing, dyspnea, and changes in bowel pattern. On presentation, the patient appeared well-hydrated and in no acute distress. Her preoperative vital signs were unremarkable, and her physical examination revealed no pertinent exam findings except for morbid obesity with a weight of 124.74 kg and body mass index (BMI) of 44.5 kg/m^2^. Resonant breath sounds were appreciated on auscultation bilaterally, and her abdomen was soft, non-tender, and without masses. Preoperative ultrasound and upper gastrointestinal (GI) series were negative for CDH. The patient successfully underwent laparoscopic sleeve gastrectomy, but an intraoperative finding of a large foramen of Morgagni was noted to be present without any herniation of GI contents. However, the hernia was not repaired due to the incidental nature of the defect and an absence of viscus herniation, strangulation, or ischemia. Postoperatively, the patient progressed well, and the remainder of her hospital course was uneventful. She was discharged home on postoperative day 1 with no developing symptoms on subsequent follow-up visits. 

## Discussion

**Figure 1:**
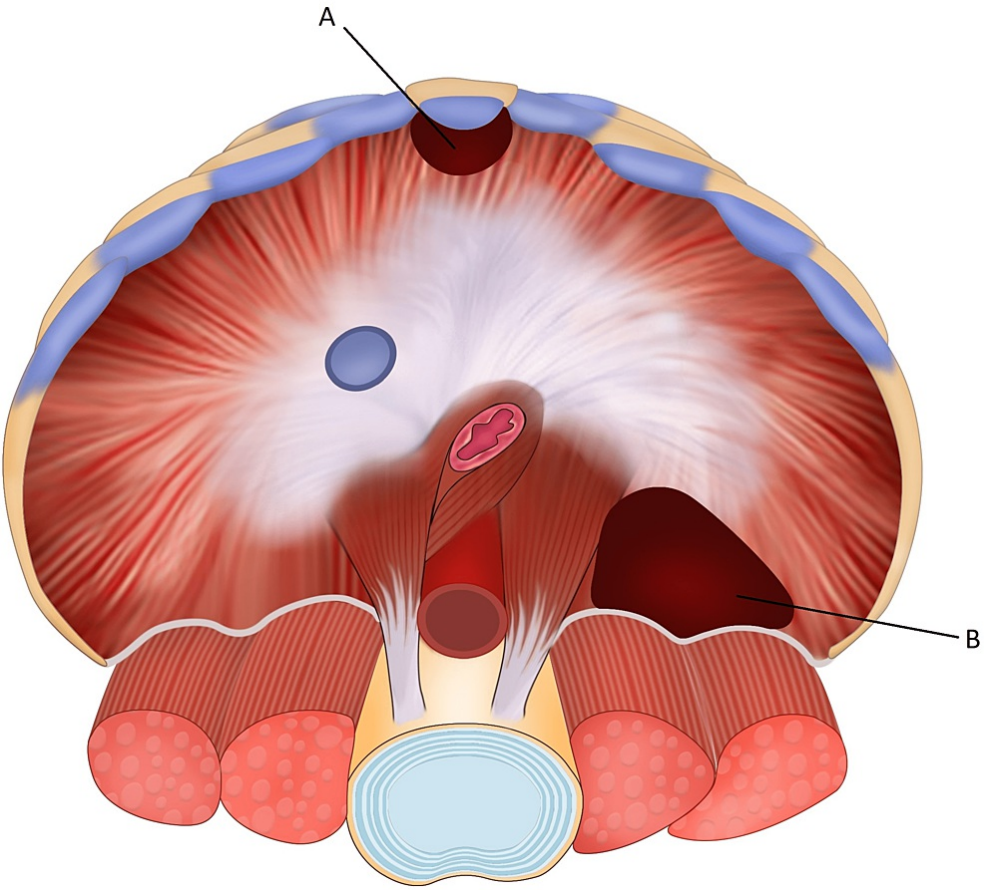
Illustration displaying the anatomical location of a Morgagni hernia (A) and a Bochdalek hernia (B). Illustration created and used with permission by our medical illustrator, Alby Joseph.

MHs identified by Giovanni-Battista Morgagni in 1769 are a rare type of CDH and are reported to occur in less than 2-3% of cases [2,3,13]. These retroxiphoid diaphragmatic defects are postulated to occur from an incomplete fusion of the septum transversum and sternum with the ribs anteriorly and are found to be right-sided in 90% of cases, left-sided in 8% of cases, and bilateral in 2% of identified cases [14] (Figure 1). Incidences have been reported more commonly in females; however, men present with symptoms earlier in life compared with women, who report symptoms more often over the age of 50 years [3]. The two most common CDH are Morgagni and Bochdalek, the latter comprising 95% of the total prevalence. Morgagni defects have also been associated with obesity and anomalies such as pentalogy of Cantrell, Tetralogy of Fallot, Turner’s syndrome, chromosomal abnormalities, and scoliosis [15-17]. Patients with Morgagni defects are typically asymptomatic early in life and often remain asymptomatic throughout their lives [10,15]. Rare complications of these hernias include strangulation, incarceration, and bowel obstruction [13]. Additionally, symptomatic patients may present with vague respiratory and GI symptoms, including cough, dyspnea, and postprandial pain [15,16]. 

Diagnostic imaging for MHs includes plain radiography, ultrasonography, computed tomography, and magnetic resonance imaging, with imaging typically notable for the presence of viscus with or without omentum herniation into the thoracic cavity [13-15]. Once identified, MHs are treated based on the acuity of patients’ presentation and the size of their hernia defect [7]. When evidence of bowel compromise exists, open transabdominal or transthoracic approaches serve as the apt management, with a preference for the former due to more satisfactory outcomes in these settings [13,16]. Transabdominal approaches are performed via subcostal, upper midline, or paramedian incisions, with midline incisions allowing for the evaluation of both sides of the hernia defect [16]. A transthoracic approach provides an excellent view for hernia repair. Although less invasive than a transabdominal approach, surgeons consider this option less favorable due to its associated morbidity and chest tube requirement [16]. Under stable conditions, these hernias can be repaired thoracoscopically or laparoscopically. Laparoscopic management is widely regarded as the gold standard in the treatment of uncomplicated hernia defects [8,13,16,18]. This approach offers excellent bilateral views, less tissue injury, shorter hospital duration, and lesser risks of perioperative and postoperative morbidity and mortality [3,16]. Thoracoscopic approaches are considered less optimal than laparoscopic management due to the limited ability to view both sides of the hernia defect [16]. 

Currently, a consensus on the management of incidental Morgagni defects during bariatric surgery is lacking in part due to the rare occurrence of these defects and limited reports addressing hernia repair in obese patients. Due to the low risk of future strangulation, Aurora et al. suggest that the need for surgical intervention is dependent upon the patient’s presentation [7]. Conversely, other studies have emphasized the importance of surgical correction of asymptomatic Morgagni defects in this setting, citing that repair avoids further complications [9,14]. Elfiky et al. reported that elderly patients diagnosed with symptomatic MH improved transiently with medical intervention with the return of symptoms, thus warranting definitive surgical repair [19]. 

To date, only a few studies describing concomitant bariatric surgery and MH repair have been reported in the literature. We identified six case reports describing such an instance [2,8-12]. Of these cases, only three reported an incidental Morgagni defect: one radiographically and two during the intraoperative period [2,9,10]. The mean age of all patients involved was 46.5 and males comprised 67% of the population. Mean BMI was 47.1 and dyspnea was the leading symptom at presentation. Radiographic evidence of MH was made in 67% of patients compared to an intraoperative diagnosis that was made in the remaining 33% of cases. Laparoscopy with mesh was the preferred operative technique for hernia repair while concomitant sleeve gastrectomy or gastric bypass was performed in the majority of cases. All patients were reported as having favorable outcomes at subsequent follow-ups. Table 1 displays a summary of key findings in patients undergoing MH repair with concomitant bariatric surgery as reported in previous studies.

**Table 1:** Summary of Key Findings in Patients Undergoing Morgagni Hernia Repair With Concomitant Bariatric Surgery as Reported in Previous Studies. BMI = body mass index; CXR = chest x-ray; CT = computed tomography; MH = Morgagni hernia; NS = not specified; RYGB = Roux-en-Y gastric bypass. *Patient previously underwent bariatric surgery for morbid obesity.

Author	No. of cases (n)	Sex	Patient age (in years)	BMI (kg/m^2^)	Presentation	Radiological diagnosis of MH?	Type of MH repair surgery performed	Type of bariatric surgery performed concurrently	Clinical outcome
Alqahtani et al. [2]	1	Female	36	66	Mild dyspnea and abdominal discomfort	Yes, incidentally via CT abdomen/pelvis	Laparoscopy with mesh	Sleeve gastrectomy	Asymptomatic at 2-month follow-up
Chiou et al. [8]	1	Male	58	48	Mild exertional dyspnea	Yes, via CT chest	Laparoscopy with mesh	Sleeve gastrectomy	Asymptomatic at 2-month follow-up
Debi et al. [9]	1	Male	53	42.6	Dyspnea	No, identified intraoperatively	None; closure obtained using non-resorbable monofilament suture	RYGB	Asymptomatic at 1-year follow-up
Kosai et al. [10]	1	Female	57	47	Asymptomatic	No, identified intraoperatively	Laparoscopy with mesh	Sleeve gastrectomy	Excess weight loss of ~ 30% with no hernia recurrence at 3-month follow-up
Li et al. [11]	1	Male	34	NS	Non-specific gastrointestinal symptoms	Yes, via CT chest	Laparoscopy with pericardium patch	None reported	Uneventful recovery
Reddy and Patel [12]	1	Male	41	32	Dyspnea and cough	Yes, via CT abdomen/pelvis	Laparoscopy with mesh	None*	Asymptomatic at 2-month follow-up

## Conclusions

While some studies propose non-operative management as an option in the setting of asymptomatic Morgagni defects, most studies support the use of surgical repair in these cases due to the potential for future incarceration and strangulation. However, guidelines on the surgical management of incidental MHs are non-existent. Presently, only a few studies describing the efficacy and safety of MH defect repairs with concomitant bariatric surgery have been reported. From this sample, there appears to be a consensus that hernia repair with or without mesh can be performed safely and effectively using a laparoscopic approach. Further review of the literature suggests that the therapeutic and rehabilitative advantages offered with laparoscopy compared with laparotomy and thoracotomy make the former technique the preferred surgical intervention for Morgagni defect repairs in patients who do undergo concomitant bariatric surgery. Due to a lack of guidelines and large-scale studies addressing the management of incidental Morgagni defects in bariatric patients, we suggest that surgeons thoroughly assess their patients’ overall clinical picture, radiographic findings, and the likelihood of future complications such as incarceration or strangulation when deciding whether to operate or observe. 
